# OP01 Evaluating Health Through The Lens Of Productivity Gains

**DOI:** 10.1017/S0266462325100603

**Published:** 2025-12-29

**Authors:** Zanfina Ademi, Dina Abushanab, Maria Arvez, Melanie Lloyd

## Abstract

**Introduction:**

To promote robust prevention strategies and reflect broader economic impacts, we developed the productivity-adjusted life years (PALYs) metric, which captures a more comprehensive view of health’s societal value. This systematic review explores the use of PALYs across various disease contexts, illustrating its application as a novel outcome measure in health economic evaluations.

**Methods:**

We conducted a comprehensive review of studies utilizing PALYs to identify effective methods for decision-making and to illustrate their applications. Using a snowball search, we selected studies that applied PALYs to quantify societal health burdens in specific diseases or contexts. Extracted data included health conditions, country setting, time frame, model type, outcomes, data type (incidence or prevalence), discounting, time horizon, prevention strategies, working-age population, productivity index components, PALY economic value, gross domestic product (GDP), and sensitivity analysis details. Income-level classifications were based on World Bank standards, and findings were summarized through narrative synthesis.

**Results:**

We reviewed 34 studies published from 2018 to 2024, covering conditions like cardiovascular and kidney disease, and environmental factors such as PM2.5 exposure. Most were conducted in high-income countries (n=20). Life table models (n=23) and dynamic models (n=8) were predominant, with other methods including mathematical projections, questionnaires, and cross-sectional designs. Studies focused more on prevention (n=18) than on chronic disease management (n=14). Diabetes, hypertension, sleep apnea, and non-optimal temperatures had the greatest societal impact, while epilepsy led to significant losses in life years and PALYs. Productivity indices varied, with losses ranging from USD1,137 to USD217,983 per full-time worker.

**Conclusions:**

The diversity of health conditions and economic contexts covered in these studies highlighted a broad interest in the determinants of health as measured by the PALY. This diversity is essential, as it provides a comprehensive understanding of the economic impacts and health challenges. Moreover, the emphasis on prevention over chronic disease management suggests a strategic shift toward averting disease onset.

